# Examining perinatal health inequities: The role of disability and risk of adverse outcomes through the U.S. Pregnancy Risk Assessment Monitoring System

**DOI:** 10.1371/journal.pone.0319950

**Published:** 2025-03-13

**Authors:** Jeanne L. Alhusen, Genevieve R. Lyons, Rosemary B. Hughes, Kathryn Laughon, Maria McDonald, Casey L. Johnson

**Affiliations:** 1 School of Nursing, University of Virginia, Charlottesville, Virginia, United States of America; 2 Department of Public Health Sciences, School of Medicine, University of Virginia, Charlottesville, Virginia, United States of America; 3 Rural Institute for Inclusive Communities, University of Montana, Missoula, Montana, United States of America; University of Bergen: Universitetet i Bergen, NORWAY

## Abstract

**Objective:**

To examine pre-pregnancy characteristics, pregnancy complications, and birth outcomes among respondents with self-reported disability compared to those without disability.

**Methods:**

A cross-sectional weighted sample of 2,006,700 respondents with singleton live births who participated in the United States Pregnancy Risk Assessment Monitoring System (PRAMS) between 2018 and 2021 provided data on disability, including difficulty in vision, hearing, ambulation, cognition, communication, and self-care. We estimated covariate-adjusted odds of differences in pre-pregnancy chronic health conditions, pregnancy intention, intimate partner violence (IPV), depression, adequacy of prenatal care, pregnancy-related health conditions, and birth outcomes by disability status.

**Results:**

Of the 2,006,700 respondents included, 59.5% reported no disability, 33.9% had moderate disability, and 6.6% had severe disability. Across most outcomes, there was a graded pattern with those with severe disability having the worst outcomes compared to the other two groups. Respondents with severe disability were more likely to report diabetes and hypertension before becoming pregnant than respondents without disabilities. Those respondents with severe disability or moderate disability had an increased odds of reporting IPV and depression than those with no disability. During pregnancy, respondents with severe disability had an increased odds of gestational diabetes (aOR 1.46, 95% CI 1.18, 1.80) and hypertensive disorders of pregnancy (aOR 1.70, 95% CI 1.43, 2.02) as compared to respondents with no disability. Respondents with moderate disability also had an increased odds of both gestational diabetes (aOR 1.19, 95% CI 1.06, 1.34) and hypertensive disorders of pregnancy (aOR 1.29, 95% CI 1.17, 1.42) as compared to those with no disability. The odds of reporting an unintended pregnancy were highest in respondents with a severe disability (aOR 1.66, 95% CI 1.43, 1.94) and were also increased in respondents with moderate disability (aOR 1.48, 95% CI 1.36, 1.62) as compared to those reporting no disability. Across most birth outcomes, respondents with severe disabilities had worse outcomes with an increased odds of low birth weight infants (aOR 1.28, 95% CI 1.08, 1.52), preterm birth (aOR 1.32, 95% CI 1.11, 1.57), and neonatal intensive care unit admission (aOR 1.45, 95% CI 1.02, 2.06) as compared to respondents with no disability. There were not differences in being classified as small for gestational age or infants’ length of hospital stay by disability status.

**Conclusions:**

Across the perinatal period, respondents with moderate or severe disability experienced worse outcomes than those without disability. There is a critical need to improve pre-conception health in an effort to reduce inequities in pregnancy outcomes. Additionally, health care providers and systems must provide equitable access to care to persons with disabilities to reduce inequities in outcomes.

## Introduction

An estimated 12-20% of U.S. persons of reproductive age have a disability [1–4]. Disabilities, while varied in their origins and effects, can be broadly categorized according to common activity limitations. Importantly, persons with disabilities are as likely to desire to become pregnant as their non-disabled peers [5]. Yet, limited literature demonstrates that pregnant and birthing persons with disabilities are at an increased risk of adverse pregnancy and neonatal outcomes including an increased risk of hypertensive disorders during pregnancy [6–8], gestational diabetes [9,10], cesarean delivery [6,11,12], low birth weight [8,13], preterm birth [8,13], and postpartum complications including hospital readmission [14] and perinatal depression [15,16]. The reasons for these disparities are multi-faceted and are, in part, due to gaps in health care provider knowledge, lack of accessibility within the health care system, and systematic barriers to education and employment leading to disproportionately high rates of preconception risk factors including obesity, diabetes, mental health conditions, and exposure to violence, including intimate partner violence (IPV) [1,17–19].

Our understanding of pregnancy-related complications and birth outcomes, across the perinatal period, at a population-based level, is limited. Analyzing the Pregnancy Risk Assessment and Monitoring System (PRAMS) data from Rhode Island, Mitra and colleagues noted significant disparities among persons with disabilities regarding health care utilization, preconception health status, pregnancy complications, and neonatal outcomes [20]. Specifically, 77.5% (95% CI 74.1, 80.6) of respondents with a disability reported receipt of prenatal care during the first trimester as compared to 83.6% (95% CI 82.8, 84.4) of respondents without a disability. Further, respondents with a disability were more likely to have preterm births (13.4%; 95% CI 11.6, 15.6 compared to 8.9%; 95% CI 8.5, 9.3 for persons without disabilities) and low birth weight neonates (10.3%; 95% CI 9.4, 11.2 compared to 6.8%; 95% CI 6.8, 6.9). Finally, respondents with a disability were significantly more likely to report vaginal bleeding, kidney or bladder infection, nausea, preterm labor, and premature rupture of membranes as compared to those without. Similarly increased risks of severe maternal morbidities and mortality were found in an analysis of the Consortium on Safe Labor data (2002-2008) which included 2074 respondents with disabilities, or 0.9% of the study sample [21].

Specifically, persons with disabilities had a higher risk of gestational diabetes, placenta previa, premature rupture of membranes, and postpartum fever than those without disabilities. Using data from the National Survey of Family Growth (2011-2019), Horner-Johnson and colleagues found respondents with disabilities had a 24% and 29% higher risk for preterm birth and low birthweight, respectively [1]. To expand our understanding of perinatal health among persons with disabilities, we examined pre-pregnancy characteristics, pregnancy complications, and birth outcomes among a national sample of U.S. respondents who recently had a live birth.

## Materials and methods

### Study population and design

We used data from the 2018–2021 waves of PRAMS, accessed January 30^th^ 2024, which links survey and birth certificate data from a national sample of postpartum respondents in the U.S. [22]. PRAMS is an ongoing state-level, population-based surveillance system of selected maternal behaviors and experiences occurring before, during, and shortly after pregnancy. Specifically, PRAMS focuses on respondents who have recently given birth to live-born infants within their respective states during the surveillance year, with sampling occurring between 2 and 6 months postpartum. Sample sizes per state vary annually, typically ranging from approximately 1000 to 3000 respondents, with determinations based on the stratification plan, number of births, and available budgets. Stratification variables that are commonly used include birth weight, maternal race and ethnicity, and Medicaid status. Data are weighted by each jurisdiction’s unique sampling approach. For each respondent, the initial sampling weight is the reciprocal of the sampling fraction applied to the stratum. For example, sampling fractions in PRAMS typically range from 1 in 1 (for low birth weight strata in smaller jurisdictions) to approximately 1 in 211 (for normal birth weight, nonminority strata in more populous jurisdictions). In this example, the corresponding sample weights would range from 1 to 211 [23].

Our sample is restricted to PRAMS sites that collected data on the variables of interest during the study period. S1 Fig (supporting information) provides a detailed breakdown of how we arrived at our final sample size. Most of these respondents (98.6%) were excluded because their respective state or batch did not ask questions pertaining to disability status. We further excluded 1530 non-singleton births and 1741 births whose plurality was not known. Thus, our weighted study population includes 2,006,700 respondents who self-reported disability status and delivered a singleton birth between 2018 and 2021. The current study was approved by the University of Virginia’s Institutional Review Board for the Social and Behavioral Sciences (Protocol #3881).

### Disability status

In 2018, a disability supplement was added to PRAMS data collection. This supplement consists of the Washington Group Short Set on Functioning (WG-SS) [24] that have been used extensively in other federal surveys. These questions are based on the World Health Organization’s International Classification of Functioning, Disability and Health and aim to provide standardized language for operationalizing disability [25]. Respondents are asked about their level of difficulty across the following domains: seeing, even when wearing glasses or contact lenses; hearing, even if using a hearing aid(s); walking or climbing steps; remembering or concentrating; completing self-care, such as washing or dressing; and communicating, understanding, or being understood in their usual language. Response options include no difficulty, some difficulty, a lot of difficulty, and cannot do this at all. We classified disability via a three-way disaggregation via degree as follows: a) respondents with no difficulty/no disability for any question, b) respondents with some difficulty/moderate disability on at least one question, and c) respondents with at least a lot of difficulty/severe disability on at least one question. This classification was done based on recent recommendations to assure we captured potential disadvantages that may vary based upon degree of functional difficulty [26]. In this paper, we will alternate between using those classifications and, for the sake of brevity, the classifications of no disability, moderate disability, and severe disability. We will also alternate between using first person language (person with a disability) and identity first language (disabled person) which is consistent with preferences in the Disability community.

### Pre-pregnancy outcomes

Our outcomes of interest were analyzed across the perinatal period with differentiation by pre-pregnancy, pregnancy, and postpartum periods which included birth outcomes. For the pre-pregnancy period, outcomes included chronic health conditions, pre-pregnancy body mass index (BMI), pregnancy intention and psychosocial stressors (i.e., depressive symptoms, IPV). Chronic health conditions assessed in PRAMS include diabetes and high blood pressure and are dichotomized as yes/no. While other conditions are assessed (i.e., allergies, asthma, sickle cell disease, anemia), we focused our analysis on those health conditions with well-established links to maternal and infant morbidity and mortality [27–30]. Self-reported anthropometric data (i.e., height, pre-pregnancy weight) is used to calculate a pre-pregnancy BMI using the standard formula for BMI. A categorical BMI variable was then created using the National Institutes of Health Heart, Lung, and Blood Institute BMI categories of underweight (<18.5 kg/m^2^), normal weight (18.5-24.9 kg/m^2^), overweight (25.0-29.9 kg/m^2^) and obese ( ≥ 30.0 kg/m^2^). Pregnancy intention is assessed via a question which asks respondents about the timing of their pregnancy and response options include they intended to be pregnant at a later time, at that time, did not want to be pregnant at that time or any time, or were not sure. Respondents who indicated they wanted to be pregnant at a later time or did not want to be pregnant at that time or any time were classified as having an unintended pregnancy [31]. PRAMS respondents are asked about depression and experiences of violence by a partner or ex-partner in the 12 months before pregnancy, during pregnancy, and in the postpartum period. Response options are dichotomized as yes/no.

### Pregnancy outcomes

During pregnancy, key outcomes included pregnancy-related health conditions (i.e., gestational diabetes, hypertensive disorder of pregnancy [HDP]), psychosocial stressors (i.e., depression, IPV) and adequacy of prenatal care. In PRAMS, HDP encompasses chronic hypertension, gestational hypertension, and preeclampsia/eclampsia and this outcome is dichotomized. Adequacy of prenatal care was assessed via the Kotelchuck Index which uses two elements obtained from birth certificate data including when prenatal care was initiated (i.e., pregnancy months 1 and 2, months 3 and 4, months 5 and 6, and months 7 to 9) and the number of prenatal visits from when prenatal care began until delivery (e.g., received services). To classify the adequacy of received services, the number of prenatal visits is compared to the expected number of visits for the period between care initiation and the delivery date. The expected number of visits is based on the American College of Obstetricians and Gynecologists prenatal care standards for uncomplicated pregnancies and is also adjusted for the gestational age when care began and for the gestational age at delivery. This is then grouped into four categories including inadequate (received less than 50% of expected visits), intermediate (50%-79%), adequate (80%-109%), or adequate plus (110% or more) [32]. Similar to the pre-pregnancy period, both IPV and depression are dichotomized based on the respondent’s experience during pregnancy.

### Postpartum complications and birth outcomes

Regarding postpartum complications and birth outcomes, key outcomes included Cesarean delivery, neonatal intensive care unit (NICU) admission, low birth weight (<2,500 grams), preterm birth (<37 completed weeks gestation), small for gestational age (birth weight less than the 10^th^ percentile of the population defined by gestational age in weeks, and adjusted for infant race/ethnicity, and sex), and length of hospital stay for the infant. All of these outcomes are dichotomized in PRAMS except length of stay, which we dichotomized using 5 days as the cutoff.

### Covariates

We selected the covariates included in our analyses a priori given their association with disability as well as perinatal health outcomes. These included maternal age (<20, 20-24, 25-34, > 34), maternal race and ethnicity (Asian, White, Black, Other/mixed, Hispanic), education level ( <high school, high school, some college [e.g., no degree, associate’s degree], college [completed at least a four-year degree]), marital status (married, other), income (<100% Federal poverty level, 101%-200% FPL, > 200% FPL), and maternal smoking status [33,34]. Maternal pre-pregnancy hypertension was adjusted for when examining risk of HDP. Models for low birth weight and preterm birth additionally adjusted for infant sex and maternal experience of IPV in the perinatal period.

### Statistical analyses

We examined differences in pre-pregnancy outcomes, pregnancy outcomes, and birth outcomes in three analyses. Prevalence rates and 95% confidence intervals were estimated. Chi-square tests with the Rao-Scott correction for complex survey design were used to test the association between outcomes and disability status. Odds ratios for binary outcomes during pregnancy and birth outcomes were modeled using multivariable logistic regression with survey weights applied. Small for gestational age (SGA) is calculated using percentiles that account for the infant’s sex and gestational age at delivery, thus we did not additionally adjust for infant sex in this model. SGA was dichotomized using the tenth percentile as cutoff.

All analyses were completed using the complex survey features of SAS v. 9.4 to apply survey weights, which account for the sampling process, design and adjusting for nonresponse and the potential for clustering around particular health care facilities, counties or time of year while also providing results that are representative of the total population of respondents who gave birth to a live infant in the states/territories and time periods under study. As birth certificate data are available for both responders and nonresponders, the information available on nonresponders is used to adjust for nonresponse as well as to understand factors associated with survey nonresponse.

## Results

Table 1 presents the weighted sample size and the weighted prevalence (with 95% confidence interval) of respondent sociodemographic, behavioral, and health condition related characteristics, via disability status categorized as no disability, moderate disability, and severe disability. In our sample, 59.5% (95% CI 58.7, 60.2) reported no difficulty in any of the domains queried, which we classified as no disability, 33.9% (95% CI 33.2, 34.6) reported having at least some difficulty on at least one question, which we classified as moderate disability, and 6.6% of respondents (95% CI 6.3, 7.0) reported at least a lot of difficulty for at least one question. These respondents were classified as having a severe disability. S1 Table provides the complete breakdown of level of disability for each type of disability assessed in PRAMS. Across all levels of disability, there were differences with respect to all socio-demographic characteristics including maternal age at delivery, race/ethnicity, household income, marital status, educational attainment, and insurance type. Specifically, respondents with disabilities tended to be younger and more likely to report a household income below the Federal poverty level.

**Table 1:** Maternal pre-pregnancy demographic, behavioral, and health condition related characteristics of PRAMS respondents who had live singleton births in 2018-2021 by disability status.

Characteristics	No disability (N = 1221123)^a^		Moderate disability (N = 696262)^a^		Severe disability (N = 135876)^a^	
	%^b^	(95% CI)^b^	%^b^	(95% CI)^b^	%^b^	(95% CI)^b^
Maternal age at delivery						
< 20	3.7	(3.3, 4.1)	4.5	(4.0, 5.1)	5.9	(4.5, 7.3)
20-24	16.7	(15.9, 17.5)	20.0	(18.9, 21.1)	26.5	(23.8, 29.2)
25-34	60.2	(59.2, 61.2)	58.7	(57.4, 60.0)	53.2	(50.2, 56.2)^c^
≥ 35	19.4	(18.6, 20.2)	16.8	(15.8, 17.7)^c^	14.4	(12.4, 16.5)^c^
Maternal race/ethnicity						
Non-Hispanic White	58.1	(57.2, 59.1)	61.4	(60.2, 62.7)^c^	56.5	(53.6, 59.5)
Non-Hispanic Black	19.5	(18.7, 20.3)	16.9	(15.9, 17.9)^c^	21.5	(19.1, 23.9)
Hispanic	13.7	(13.0, 14.4)	13.7	(12.8, 14.6)	13.8	(11.6, 16.0)
Other	8.7	(8.1, 9.2)	8.0	(7.3, 8.6)	8.2	
Maternal education						
Some high school education or less	10.9	(10.2, 11.4)	10.5	(9.7, 11.3)	17.7	(15.2, 20.1)^c^
High school graduate	23.4	(22.5, 24.3)	27.8	(26.5, 29.0)^c^	37.3	(34.4, 40.3)^c^
Some college education	23.5	(22.7, 24.3)	29.5	(28.4, 30.7)^c^	30.4	(27.9, 33.0)^c^
College graduate or more	42.2	(41.2, 43.2)	32.2	(31.0, 33.4)^c^	14.5	(12.4, 16.6)^c^
Household income^d^						
< 100% Federal poverty level (FPL)	24.6	(23.7, 25.5)	30.7	(29.5, 32.0)^c^	50.7	(47.6, 53.9)^c^
101%-200%FPL	20.6	(19.7, 21.5)	25.2	(24.0, 26.3)^c^	25.1	(22.4, 27.7)^c^
> 200% FPL	54.8	(53.8, 55.8)	44.1	(42.8, 45.5)^c^	24.2	(21.6, 26.8)^c^
Marital status						
Married	64.6	(63.6, 65.6)	57.4	(56.1, 58.7)^c^	41.3	(38.4, 44.4)^c^
Other	35.4	(34.4, 36.4)	42.6	(41.3, 43.9)^c^	58.6	(55.6, 61.6)^c^
Health insurance						
Medicaid	38.0	(37.0, 39.0)	43.8	(42.5, 45.1)^c^	62.5	(59.6, 65.5)^c^
Private	55.0	(54.0, 56.0)	49.1	(47.7, 50.4)^c^	31.2	(28.4, 33.9)^c^
Other	7.0	(6.4, 7.6)	7.2	(6.4, 7.9)	6.3	(4.7, 7.8)
Pre-pregnancy BMI						
Underweight (<18.5 kg/m^2^)	8.0	(7.5, 8.6)	8.0	(7.2, 8.7)	7.9	(6.4, 9.5)
Normal (18.5-24.9 kg/m^2^)	47.0	(46.0, 48.1)	42.3	(40.9, 43.6)^c^	37.5	(34.5, 40.4)^c^
Overweight (25-29.9 kg/m^2^)	15.1	(14.4, 15.9)	15.4	(14.4, 16.3)	14.0	(11.9, 16.1)
Obese (≥30 kg/m^2^)	29.8	(28.9, 30.7)	34.4	(33.2, 35.7)^c^	40.6	(37.6, 43.6)^c^
Chronic health conditions, pre-pregnancy						
Type I or II Diabetes	2.9	(2.5, 3.2)	2.7	(2.3, 3.1)	4.7	(3.4, 6.0)
High blood pressure	4.6	(4.2, 5.0)	5.3	(4.8, 5.9)	8.3	(6.8, 9.8)
Depression	9.0	(8.4, 9.5)	24.1	(23.0, 25.2)	44.1	(41.1, 47.1)
Smoked during last 3 months of pregnancy	4.7	(4.3, 5.2)	8.8	(8.1, 9.5)	19.5	(17.0, 21.9)
Psychosocial stressors						
Intimate partner violence before pregnancy	1.4	(1.1, 1.6)	4.1	(3.6, 4.7)^c^	9.4	(7.8, 11.1)^c^
Intimate partner violence during pregnancy	1.1	(0.9, 1.3)	2.6	(2.2, 3.0)^c^	6.0	(4.6, 7.4)^c^
Pregnancy Intendedness						
Unintended pregnancy	21.2	(20.4, 22.1)	28.9	(27.7, 30.1)^c^	34.4	(31.6, 37.3)^c^
Not unintended pregnancy	78.8	(77.9, 79.6)	71.1	(69.9, 72.3)^c^	65.6	(62.7, 68.4)^c^

^a^Weighted sample size.

^b^Weighted prevalence and corresponding 95% confidence intervals (expressed as percentage).

^c^Indicates that the group estimate differs from reference level (no disability).

^d^FPL based upon income and household size. Income was reported in ranges. Respondents’ %FPL calculated using the midpoint of their reported income range and reported number of dependents.

With regards to pre-pregnancy chronic health conditions, respondents with severe disability were more likely to report diabetes compared to those without any difficulty/no disability (4.7% vs. 2.9%, respectively), as well as high blood pressure (8.3% versus 4.6%, respectively). Smoking was higher in those with severe disability (19.5%, 95% CI 17.0, 21.9) as compared to 4.7% (95% CI 4.3, 5.2) for respondents without disability. Differences in pregnancy intention, depressive symptoms, and IPV status also varied by disability status with those with severe disability having the highest risk (see Table 1).

Table 2 outlines adequacy of pregnancy care, pregnancy-related health conditions, and infant outcomes. Adequacy of prenatal care, as measured by the Kotelchuck index, varied by disability level with respondents with severe disability more likely to report inadequate prenatal care as compared to respondents without a disability (16.6% vs. 11.9%, respectively). With respect to pregnancy-related health conditions, respondents with severe disability had the highest prevalence of both gestational diabetes and HDP. Infants born to respondents with severe disability were more likely to be born preterm, low birthweight, and require NICU admission than infants born to respondents without a disability (see Table 2).

**Table 2:** Pregnancy care and infant outcomes of PRAMS respondents who had live singleton births in 2018-2021 by disability status.

Characteristics	No disability (N = 1221123)^a^		Moderate disability (N = 696262)^a^		Severe disability (N = 135876)^a^		p value^c^
	No^a^	%(95% CI)^b^	No^a^	%(95% CI)^b^	No^a^	%(95% CI)^b^	
Gestational diabetes							
Yes	9.9	(9.0, 10.2)	10.8	(10.0, 11.5)	13.2	(10.7,15.8)	0.002
Hypertensive disorder of pregnancy							
Yes	10.4	(9.8, 11.0)	11.2	(10.4, 11.9)	13.3	(10.9, 15.6)	0.018
Preterm birth (<37 completed weeks gestation)							
Yes	8.3	(7.9, 8.7)	8.7	(8.1, 9.3)	11.2	(9.7, 12.7)	<0.001
Low birth weight (<2,500 grams)							
Yes	6.5	(6.1, 6.8)	7.1	(6.6, 7.6)	9.2	(7.8,10.6)	<0.001
Small for gestational age							
Yes	9.7	(9.2, 10.3)	10.3	(9.5, 11.0)	9.6	(8.0, 11.2)	0.51
Neonatal intensive care unit admission							
Yes	11.8	(10.4, 13.2)	13.4	(11.6, 15.2)	17.1	(12.7, 21.4)	0.04
Infant stayed in hospital > 5 days							
Yes	1.4	(1.2, 1.6)	1.4	(1.1, 1.7)	0.9	(0.4, 1.3)	0.30
Delivery method							
Vaginal	66.0	(65.0, 66.9)	67.3	(66.1, 68.6)	64.4	(61.5, 67.2)	0.11
Assisted	3.3	(2.9, 3.7)	2.7	(2.3, 3.2)	3.7	(2.4, 5.1)	
C-section	30.7	(29.8, 31.7)	30.0	(28.8, 31.1)	31.9	(29.1, 34.6)	
Adequacy of Prenatal Care (Kotelchuck Index)							
Inadequate	11.9	(11.2, 12.6)	12.1	(11.3, 13.0)	16.6	(14.4, 18.9)	<0.001
Intermediate	10.6	(10.0, 11.2)	10.4	(9.6, 11.2)	11.7	(9.7, 13.6)	
Adequate	46.1	(45.1, 47.1)	43.6	42.3, 44.9)	38.9	(35.9, 41.8)	
Adequate plus	31.4	(30.5, 32.4)	33.9	32.6, 35.1)	32.8	(30.0, 35.7)	

^a^Weighted sample size.

^b^Weighted prevalence and corresponding 95% confidence intervals (expressed as percentage).

^c^p-values represent differences across level of disability.

Table 3 presents the adjusted odds ratios for key maternal and infant health outcomes, comparing respondents with no disability or moderate disability to those with severe disability. Refer to S2 Table for full details of all covariates in these models.

**Table 3:** Adjusted odds ratios compared against no disability (reference group) for maternal and birth outcomes by disability status^a^.

Outcome	aOR^b^	(95% CI)^c^
Maternal diabetes, pre-pregnancy		
Moderate disability	0.97	(0.79, 1.18)
Severe disability	1.73	(1.25, 2.40)
Maternal hypertension, pre-pregnancy		
Moderate disability	1.17	(1.01, 1.36)
Severe disability	1.79	(1.41, 2.27)
Unintended pregnancy		
Moderate disability	1.48	(1.36, 1.62)
Severe disability	1.66	(1.43, 1.94)
Gestational diabetes		
Moderate disability	1.19	(1.06, 1.34)
Severe disability	1.46	(1.18, 1.80)
Hypertensive disorders of pregnancy		
Moderate disability	1.29	(1.17, 1.42)
Severe disability	1.70	(1.43, 2.02)
IPV		
Moderate disability	2.22	(1.71, 2.90)
Severe disability	4.22	(3.02, 5.90)
Depression		
Moderate disability	3.26	(2.94, 3.61)
Severe disability	7.54	(6.43, 8.84)
Inadequate vs adequate/adequate+		
Moderate disability	0.99	(0.91,1.08)
Severe disability	1.20	(1.03,1.41)
Low birth weight		
Moderate disability	1.08	(0.97, 1.20)
Severe disability	1.28	(1.08, 1.52)
Preterm birth		
Moderate disability	1.08	(0.97, 1.19)
Severe disability	1.32	(1.11, 1.57)
Small for gestational age		
Moderate disability	1.00	(0.89, 1.12)
Severe disability	0.84	(0.68, 1.05)
NICU admission		
Moderate disability	1.15	(0.92, 1.43)
Severe disability	1.45	(1.02, 2.06)
5+ day hospital stay		
Moderate disability	0.96	(0.72, 1.28)
Severe disability	0.75	(0.41, 1.36)

^a^Models adjusted for maternal age, race/ethnicity, marital status, education level, income status and maternal smoking status. LBW and PTB also adjusted for infant sex and maternal IPV.

^b^aOR =  adjusted odds ratio.

^c^95% CI = 95% Confidence Interval.

With regards to maternal chronic health conditions, respondents with severe disability had 1.7 times the odds of being diagnosed with diabetes than those with no disability (aOR 1.73, 95% CI 1.25, 2.40) as well as 1.8 times the odds of being diagnosed with hypertension compared to respondents with no disability (aOR 1.79, 95% CI 1.41, 2.27). Additionally, the odds of hypertension were also increased in respondents with moderate disability as compared to respondents with no disability (aOR 1.17, 95% CI 1.01, 1.36).

An examination of pregnancy-related health conditions demonstrated that the odds of being diagnosed with gestational diabetes were higher in respondents with severe disability (aOR 1.46, 95% CI 1.18, 1.80) and those with moderate disability (aOR 1.19, 95% CI 1.06, 1.34) as compared to respondents with no disability. Similar differences were noted when examining HDP. We found that respondents with severe disability had the highest odds (aOR 1.70, 1.43, 2.02) of being diagnosed with HDP, followed by respondents with moderate disability (aOR 1.29, 95% CI 1.17, 1.42) as compared to those with no disability. An analysis of psychosocial stressors revealed respondents with severe disability had 4.2 the odds (aOR 4.22, 95% CI 3.02, 5.90) of experiencing IPV during pregnancy and those with moderate disability had 2.2 the odds (aOR 2.22, 95% CI 1.71, 2.90) of experiencing IPV during pregnancy as compared to respondents with no disability. The odds of depression during pregnancy was highest in respondents with severe disability (aOR 7.54, 95% CI 6.43, 8.84) while respondents with moderate disability had 3.3 the odds (aOR 3.26, 2.94, 3.61) of reporting depression during pregnancy as compared to respondents with no disability. Finally, respondents with severe disability had 1.6 the odds (aOR 1.66, 95% CI 1.43, 1.94) of reporting an unintended pregnancy and respondents with moderate disability had 1.5 times the odds (aOR 1.48, 95% CI 1.36, 1.62) of reporting an unintended pregnancy as compared to respondents with no disability.

When analyzing birth outcomes, we observed that respondents who reported having severe disability had 1.3 the odds (aOR 1.28, 95% CI 1.08, 1.52) of low birth weight compared to respondents reporting no disability while there were not differences in low birth weight when comparing respondents with moderate disability to those without a disability. Similarly for preterm birth, we observed respondents reporting severe disability had 1.3 the odds (aOR 1.32, 95% CI 1.11, 1.57) of preterm birth as compared to those with no disability. The odds of an infant being born small for gestational age did not vary by disability status nor did an infant’s risk of staying in the hospital after birth for more than five days. However, infants born to respondents with severe disability had nearly 1.5 times the odds of being admitted to the NICU as compared to infants born to respondents with no disability (aOR 1.45, 95% CI 1.02, 2.06). Similar to other birth outcomes, differences were not noted when comparing odds of NICU admission for infants born to respondents with moderate disability as compared to those born to respondents with no disability.

## Discussion

This population-based study expands the understanding of perinatal health in the context of disability by showing significant disparities between persons with disability and those without disability on important predictors of maternal and infant health outcomes. In covariate-adjusted odds ratios, respondents with disabilities had higher odds of entering pregnancy with chronic health conditions (i.e., diabetes, hypertension), experiencing adverse pregnancy-related health conditions (i.e., gestational diabetes, hypertensive disorders of pregnancy), reporting depressive symptoms and experiences of IPV, and adverse birth outcomes including low birth weight, preterm birth, and NICU admission. Importantly, respondents with disability were more likely to be socially disadvantaged in several characteristics associated with increased maternal morbidity including lower income, poverty [18,35,36], less education [18,36], unmarried status [21], and use of public insurance [21]. Notably, most of the severely disabled respondents had a household income below the poverty level compared to about one quarter of non-disabled respondents. This finding is particularly salient since living with disability can place significant economic strain on people to meet disability-related needs of health care, adaptive equipment, assistive technologies, accessible transportation, and care provider services.

Across all measures of preconception health, respondents with the most severe disabilities had the highest risks. Our observation of higher prevalence of obesity, diabetes, and high blood pressure in respondents with severe disabilities may reflect the unique disability-related risks for these health conditions including limited resources for healthy diet, medications causing weight and appetite changes, limited ability to exercise, lack of accessible environments and facilities for physical activity and greater barriers to receiving high-quality health care [37]. Our finding that the odds of unintended pregnancy was higher in respondents with disability, especially those with severe disability, as compared to non-disabled respondents is consistent with other research comparing differences between disabled and nondisabled respondents [38]. Reducing the risk of unintended pregnancy is an important public health issue given its association with inadequate prenatal care, increased risk of substance use during pregnancy as well as its association with adverse birth outcomes, including preterm birth and low birthweight infants [39,40]. Further, and particularly relevant to persons with disabilities, the ability to freely determine whether and when they have children is central to reproductive autonomy. Persons with disabilities have endured a long history of marginalization, particularly as it relates to sexual and reproductive health and rights. Taken together, our results highlight the critical role of prepregnancy care for persons with disabilities which provides an opportunity for health care providers to work with pregnant persons to optimize their health, address and reduce modifiable risk factors while also providing education about a healthy pregnancy. The American College of Obstetricians and Gynecologists recommends that prepregnancy counseling should occur several times during a woman’s reproductive lifespan [41]. For persons with disabilities who do not want to become pregnant in the next year, health care providers are ideally positioned to assure they have access to appropriate contraceptive choices. Prepregnancy care also provides an opportunity to assess and intervene on psychosocial risks, including depression and violence, both known to be associated with adverse maternal and infant outcomes [42,43]. We were not able to assess prepregnancy care via PRAMS, though this is an important area for future research.

Our finding of an increased odds of IPV in disabled respondents is consistent with other violence research [17,44–47]. Our findings are novel in that prior research has largely used community-based samples or a dichotomized measure of disability. It stands to reason that people with severe disability would also experience the most IPV given their likely dependence on abusive partners or caregivers for financial resources and assistance with daily survival activities such as eating, hygiene, and access to medications as well as multiple barriers to escaping IPV [48]. As PRAMS assessment of IPV is limited to physical abuse, an important area for additional research includes a more comprehensive understanding of the various types of abuse experienced by pregnant persons with disability. Infants born to respondents with the most severe level of disability had the highest risk of being born preterm or low birth weight. Diabetes (type 1 and 2), gestational diabetes, and obesity are known risk factors for preterm birth [49,50]. Preterm birth is associated with an increased risk of infant morbidity, including chronic lung disease, retinopathy of prematurity, and necrotizing enterocolitis [51,52]. While we were not able to assess infant morbidity beyond NICU admission or ≥ 5 day length of hospital stay, an enhanced understanding of the associations between maternal disability and infant morbidity is important in guiding pre-conception and pregnancy care. An additional risk factor for preterm birth and low birth weight is IPV, a risk factor substantiated in a relatively recent meta-analysis of 50 studies [53]. Experiencing IPV during pregnancy may lead to adverse birth outcomes via several pathways including direct trauma to the abdomen or sexual assault which may lead to premature rupture of membranes or placental damage [53]. Further, women experiencing IPV may have increased behavioral risk factors during pregnancy (e.g., smoking, substance use, inadequate prenatal care) that are associated with adverse birth outcomes [42,54]. Future research should focus on different types of violence experienced by persons with disabilities and the potential pathways by which IPV may influence birth outcomes.

Given our findings of increased risk of diabetes and gestational diabetes for respondents with the most severe disabilities, future research should examine glycemic control and infant status, including NICU admission, as this is a risk factor that could be reduced with appropriate counseling and monitoring. Currently, PRAMS does not allow the distinction between type 1 and type 2 diabetes, nor does it provide information on the level of glycemic control before or during pregnancy.

A strength of this analysis is the use of the large, representative PRAMS dataset which includes multiple well-validated measures relevant to our research questions. Limitations of our analysis include several factors inherent to retrospective analyses of existing data. First, the PRAMS dataset includes only individuals who had a live birth. Disability status may be linked to fetal demise, and thus bias our findings. Because not all states implemented the disability supplement, our analysis is representative only of the 22 states and District of Columbia included here, although we compared the characteristics of our sample to those of the full population and noted no major differences. PRAMS relies on self-report and is therefore subject to recall bias, comprehension errors, and non-response. Additionally, birth outcomes were obtained from birth certificates and not validated by medical records. Use of the WG-SS questions to measure disability introduces both strengths and limitations. The WG-SS is a well-validated instrument that allows for comparison across multiple studies and cross-nationally [55]. Notably, it measures the ways that functional impairments interact with environmental barriers rather than relying on a medical diagnosis. Classifying respondents in three categories of no, moderate and severe disability allowed us to examine how outcomes of interest varied according to the degree of functional difficulty. However, the WG-SS may not adequately capture all types of disabilities, particularly those with less severe disabilities [56].

Our findings underscore the need to improve prepregnancy care to this growing population who are as likely to desire children as those without disabilities [5]. Delayed or inadequate prenatal care, likely shaped by social disadvantage, highlights the need for a systematic approach to addressing these risks. Factors such as socioeconomic status, educational opportunities, employment options, and housing stability impact the health outcomes of mothers and infants, and decades of research point an embarrassing finger at the inequities experienced by disabled populations. By addressing these underlying social risks, we can create environments that promote healthier pregnancies thereby reducing maternal and infant health inequities.

## Supporting information

S1 Table Breakdown of disability type and level of disability. (DOCX)

S2 Table Full model results for maternal and infant health outcomes by level of disability. (DOCX)

S1 Fig Respondent inclusion flow chart. (TIF)
