# Supplementary material for: Examining perinatal health inequities: The role of disability and risk of adverse outcomes through the U.S. Pregnancy Risk Assessment Monitoring System
Source: PLoS One. 2025 Mar 13;20(3):e0319950. doi: 10.1371/journal.pone.0319950 (PMC11906042; doi:10.1371/journal.pone.0319950)
Supplement: S1 Table — (DOCX) [file pone.0319950.s001.docx]

Supplemental Table 1: Breakdown of Disability Type and Level of Difficulty

17% (16.4, 17.5) of respondents report some difficulty seeing.

4.1% (3.8, 4.4) of respondents report some difficulty hearing

5.1% (4.7, 5.4) report some difficulty walking

24.2% (23.6, 24.9) report some difficulty remembering

2.2% (2.0, 2.4) report some difficulty with self-care

4.2% (3.9, 4.5) report some difficulty with communicating

| Type of Disability |  | Frequency | Weighted Frequency |  | Percent |  | 95% Confidence Limits for Percent | |
| --- | --- | --- | --- | --- | --- | --- | --- | --- |
| Seeing | NO DIFFICULTY | 35980 | 1665885 |  | 81.2 |  | 80.6 | 81.8 |
|  | SOME DIFFICULTY | 7520 | 348150 |  | 17.0 |  | 16.4 | 17.5 |
|  | LOT OF DIFFICULTY | 803 | 31994 |  | 1.6 |  | 1.4 | 1.7 |
|  | CANNOT DO | 155 | 5378 |  | 0.3 |  | 0.2 | 0.3 |
|  |  |  |  |  |  |  |  |  |
| Hearing | NO DIFFICULTY | 42280 | 1953184 |  | 95.2 |  | 94.9 | 95.6 |
|  | SOME DIFFICULTY | 1840 | 84371 |  | 4.1 |  | 3.8 | 4.4 |
|  | LOT OF DIFFICULTY | 188 | 8107 |  | 0.4 |  | 0.3 | 0.5 |
|  | CANNOT DO | 151 | 5155 |  | 0.3 |  | 0.2 | 0.3 |
|  |  |  |  |  |  |  |  |  |
|  |  |  |  |  |  |  |  |  |
| Walking | NO DIFFICULTY | 41747 | 1933401 |  | 94.2 |  | 93.9 | 94.6 |
|  | SOME DIFFICULTY | 2370 | 104244 |  | 5.1 |  | 4.7 | 5.4 |
|  | LOT OF DIFFICULTY | 281 | 11484 |  | 0.6 |  | 0.5 | 0.7 |
|  | CANNOT DO | 72 | 2719 |  | 0.1 |  | 0.1 | 0.2 |
|  |  |  |  |  |  |  |  |  |
| Remembering | NO DIFFICULTY | 31890 | 1474391 |  | 71.9 |  | 71.2 | 72.5 |
|  | SOME DIFFICULTY | 10811 | 497352 |  | 24.2 |  | 23.6 | 24.9 |
|  | LOT OF DIFFICULTY | 1702 | 77151 |  | 3.8 |  | 3.5 | 4.1 |
|  | CANNOT DO | 71 | 3077 |  | 0.2 |  | 0.1 | 0.2 |
|  |  |  |  |  |  |  |  |  |
| Self Care | NO DIFFICULTY | 43255 | 2000053 |  | 97.5 |  | 97.2 | 97.7 |
|  | SOME DIFFICULTY | 1049 | 44766 |  | 2.2 |  | 2.0 | 2.4 |
|  | LOT OF DIFFICULTY | 107 | 5368 |  | 0.3 |  | 0.2 | 0.3 |
|  | CANNOT DO | 62 | 1623 |  | 0.1 |  | 0.0 | 0.1 |
|  |  |  |  |  |  |  |  |  |
| Communicating | NO DIFFICULTY | 41861 | 1953091 |  | 95.2 |  | 94.9 | 95.5 |
|  | SOME DIFFICULTY | 2250 | 85605 |  | 4.2 |  | 3.9 | 4.5 |
|  | LOT OF DIFFICULTY | 285 | 10844 |  | 0.5 |  | 0.4 | 0.6 |
|  | CANNOT DO | 71 | 2347 |  | 0.1 |  | 0.1 | 0.2 |
|  |  |  |  |  |  |  |  |  |
