# Supplementary material for: Examining perinatal health inequities: The role of disability and risk of adverse outcomes through the U.S. Pregnancy Risk Assessment Monitoring System
Source: PLoS One. 2025 Mar 13;20(3):e0319950. doi: 10.1371/journal.pone.0319950 (PMC11906042; doi:10.1371/journal.pone.0319950)
Supplement: S2 Table — (DOCX) [file pone.0319950.s002.docx]

Supplemental Table 2: Full Model Results for Maternal and Infant Health Outcomes by Level of Disability

| Model outcome | Covariate | Point Estimate | 95% Confidence Limits | | p-value |
| --- | --- | --- | --- | --- | --- |
| Low Birthweight | Severe Disability vs No Disability | 1.282 | 1.084 | 1.515 | 0.0036 |
|  | Moderate Disability vs No Disability | 1.079 | 0.970 | 1.201 | 0.1631 |
|  | Maternal Age 20 - 24 vs 25-34 | 1.095 | 0.955 | 1.256 | 0.1938 |
|  | Maternal Age 35 + vs 25-34 | 1.096 | 0.865 | 1.387 | 0.4479 |
|  | Maternal Age < 20 vs 25-34 | 1.344 | 1.187 | 1.522 | <.0001 |
|  | Smoking Yes vs No | 1.834 | 1.578 | 2.132 | <.0001 |
|  | Black non-Hispanic vs White non-Hispanic | 2.301 | 2.047 | 2.587 | <.0001 |
|  | Hispanic vs White non-Hispanic | 1.100 | 0.931 | 1.301 | 0.2633 |
|  | Other/Mixed Race vs White non-Hispanic | 1.546 | 1.303 | 1.833 | <.0001 |
|  | Education < High School vs Bachelors + | 1.354 | 1.125 | 1.629 | 0.0013 |
|  | Education High School vs Bachelors + | 1.513 | 1.303 | 1.756 | <.0001 |
|  | Education Some college vs Bachelors + | 1.249 | 1.097 | 1.422 | 0.0008 |
|  | Infant Sex Female vs Male | 1.320 | 1.198 | 1.453 | <.0001 |
|  | IPV before or during pregnancy, Yes vs No | 1.443 | 1.143 | 1.822 | 0.0020 |
|  |  |  |  |  |  |
| Preterm Birth | Severe Disability vs No Disability | 1.321 | 1.111 | 1.571 | 0.0016 |
|  | Moderate Disability vs No Disability | 1.076 | 0.971 | 1.193 | 0.1620 |
|  | Maternal Age 20 - 24 vs 25-34 | 0.994 | 0.745 | 1.325 | 0.9655 |
|  | Maternal Age 35 + vs 25-34 | 1.003 | 0.880 | 1.143 | 0.9693 |
|  | Maternal Age < 20 vs 25-34 | 1.367 | 1.211 | 1.543 | <.0001 |
|  | Smoking Yes vs No | 1.418 | 1.187 | 1.694 | 0.0001 |
|  | Black non-Hispanic vs White non-Hispanic | 1.738 | 1.552 | 1.945 | <.0001 |
|  | Hispanic vs White non-Hispanic | 0.936 | 0.802 | 1.093 | 0.4055 |
|  | Other/Mixed Race vs White non-Hispanic | 1.319 | 1.113 | 1.561 | 0.0013 |
|  | Education < High School vs Bachelors + | 1.288 | 1.065 | 1.557 | 0.0090 |
|  | Education High School vs Bachelors + | 1.392 | 1.209 | 1.603 | <.0001 |
|  | Education Some college vs Bachelors + | 1.259 | 1.113 | 1.423 | 0.0002 |
|  | Infant Sex Female vs Male | 0.863 | 0.785 | 0.948 | 0.0022 |
|  | IPV before or during pregnancy, Yes vs No | 1.260 | 1.007 | 1.577 | 0.0431 |
|  |  |  |  |  |  |
| SGA-10 | Severe Disability vs No Disability | 0.84 | 0.68 | 1.05 | 0.1238 |
|  | Moderate Disability vs No Disability | 1.00 | 0.89 | 1.12 | 0.9550 |
|  | Maternal Age 20 - 24 vs 25-34 | 1.16 | 1.00 | 1.35 | 0.0516 |
|  | Maternal Age 35 + vs 25-34 | 0.97 | 0.84 | 1.11 | 0.6398 |
|  | Maternal Age < 20 vs 25-34 | 1.28 | 0.98 | 1.67 | 0.0719 |
|  | Smoking Yes vs No | 1.81 | 1.52 | 2.15 | <.0001 |
|  | Race: Black non-Hispanic vs White non-Hispanic | 0.92 | 0.79 | 1.07 | 0.2842 |
|  | Hispanic vs White non-Hispanic | 0.94 | 0.79 | 1.12 | 0.4927 |
|  | Other/Mixed Race vs White non-Hispanic | 1.09 | 0.91 | 1.31 | 0.3527 |
|  | Education < High School vs Bachelors + | 1.29 | 1.05 | 1.58 | 0.0165 |
|  | Education High School vs Bachelors + | 1.40 | 1.19 | 1.65 | <.0001 |
|  | Education Some college vs Bachelors + | 0.97 | 0.84 | 1.11 | 0.6242 |
|  |  |  |  |  |  |
| NICU Stay | Severe Disability vs No Disability | 1.45 | 1.02 | 2.06 | 0.0395 |
|  | Moderate Disability vs No Disability | 1.15 | 0.92 | 1.43 | 0.2148 |
|  | Maternal Age 20 - 24 vs 25-34 | 1.22 | 0.94 | 1.59 | 0.1429 |
|  | Maternal Age 35 + vs 25-34 | 1.66 | 1.26 | 2.20 | 0.0004 |
|  | Maternal Age < 20 vs 25-34 | 1.54 | 1.01 | 2.33 | 0.0433 |
|  | Smoking Yes vs No | 1.18 | 0.81 | 1.70 | 0.3954 |
|  | Race: Black non-Hispanic vs White non-Hispanic | 1.02 | 0.81 | 1.29 | 0.8526 |
|  | Hispanic vs White non-Hispanic | 1.34 | 0.85 | 2.13 | 0.2108 |
|  | Other/Mixed Race vs White non-Hispanic | 1.37 | 0.93 | 2.01 | 0.1114 |
|  | Education < High School vs Bachelors + | 1.40 | 0.94 | 2.08 | 0.0977 |
|  | Education High School vs Bachelors + | 1.49 | 1.08 | 2.07 | 0.0155 |
|  | Education Some college vs Bachelors + | 1.36 | 1.02 | 1.81 | 0.0351 |
|  |  |  |  |  |  |
| 5+ day Hospital stay | Severe Disability vs No Disability | 0.75 | 0.41 | 1.36 | 0.3361 |
|  | Moderate Disability vs No Disability | 0.96 | 0.72 | 1.28 | 0.7746 |
|  | Maternal Age 20 - 24 vs 25-34 | 0.65 | 0.43 | 0.98 | 0.0376 |
|  | Maternal Age 35 + vs 25-34 | 1.77 | 1.30 | 2.42 | 0.0003 |
|  | Maternal Age < 20 vs 25-34 | 0.17 | 0.08 | 0.39 | <.0001 |
|  | Smoking Yes vs No | 0.24 | 0.14 | 0.43 | <.0001 |
|  | Race: Black non-Hispanic vs White non-Hispanic | 0.37 | 0.24 | 0.56 | <.0001 |
|  | Hispanic vs White non-Hispanic | 0.26 | 0.15 | 0.44 | <.0001 |
|  | Other/Mixed Race vs White non-Hispanic | 0.38 | 0.24 | 0.60 | <.0001 |
|  | Education < High School vs Bachelors + | 4.17 | 2.40 | 7.26 | <.0001 |
|  | Education High School vs Bachelors + | 0.88 | 0.61 | 1.29 | 0.5159 |
|  | Education Some college vs Bachelors + | 1.18 | 0.84 | 1.66 | 0.3373 |
