# Supplementary figures and images for: Examining perinatal health inequities: The role of disability and risk of adverse outcomes through the U.S. Pregnancy Risk Assessment Monitoring System
Source: PLoS One. 2025 Mar 13;20(3):e0319950. doi: 10.1371/journal.pone.0319950 (PMC11906042; doi:10.1371/journal.pone.0319950)

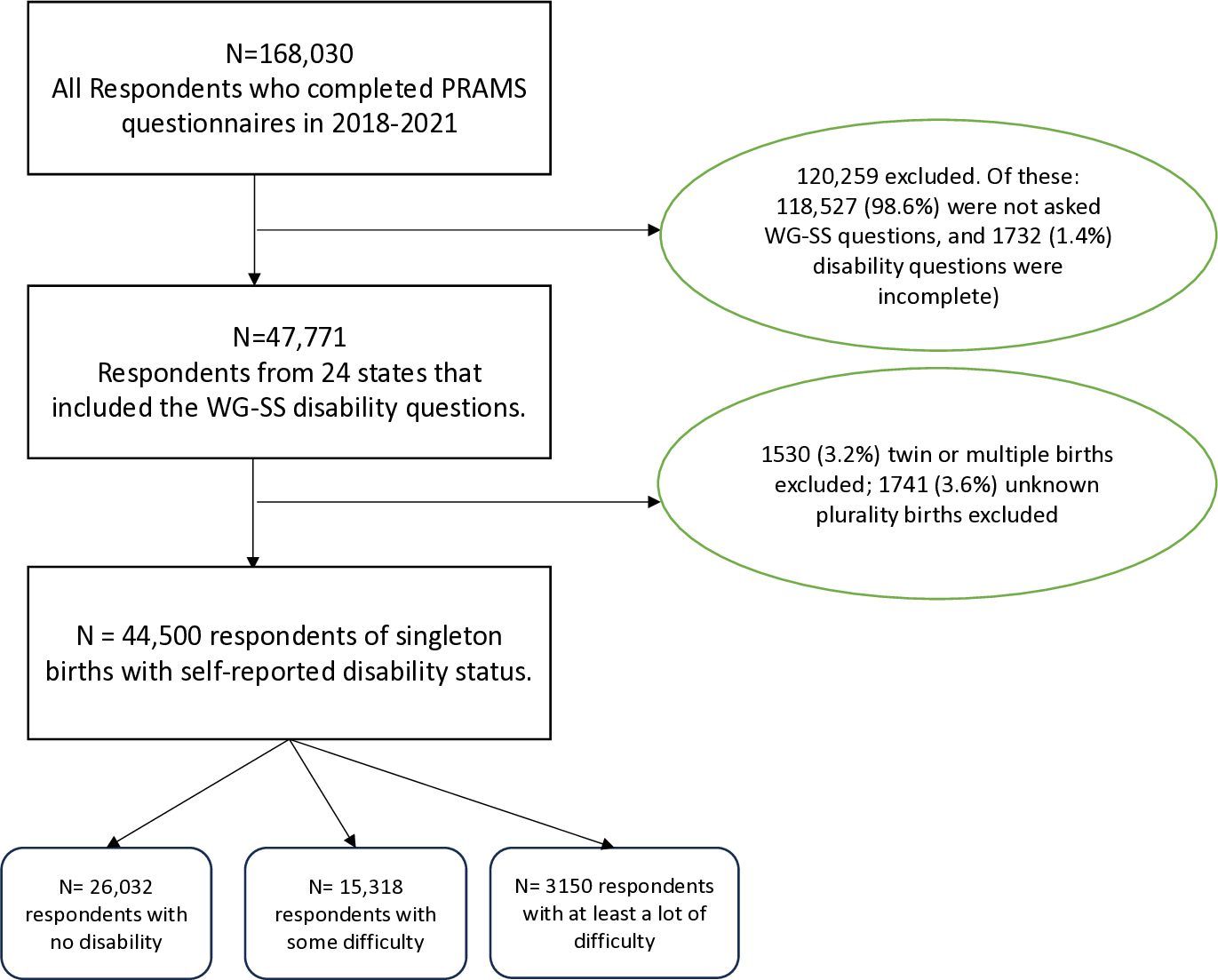

Supplement: S1 Fig — (TIF) [file pone.0319950.s003.tif]
